# Seroprevalence of hepatitis C virus among people living with HIV/AIDS in Latin America and the Caribbean: a systematic review

**DOI:** 10.1186/s12879-016-1988-y

**Published:** 2016-11-09

**Authors:** Fatima Mitiko Tengan, Karim Yakub Ibrahim, Bianca Peixoto Dantas, Caroline Manchiero, Mariana Cavalheiro Magri, Wanderley Marques Bernardo

**Affiliations:** 1 Department of Infectious and Parasitic Diseases, School of Medicine, University of São Paulo (Universidade de São Paulo - USP), São Paulo, Brazil; 2 Laboratory of Viral Medical Research in Hepatology (Laboratório de Investigação Médica em Hepatologia por vírus - LIM-47), Clinical Hospital, School of Medicine, USP, São Paulo, Brazil; 3 School of Medicine, USP, São Paulo, Brazil; 4 Brazilian Medical Association, São Paulo, Brazil

**Keywords:** Hepatitis C, HCV, HIV, Coinfection, Seroprevalence, Latin America, Caribbean

## Abstract

**Background:**

Studies have shown that the immunosuppression induced by the human immunodeficiency virus (HIV) accelerates the natural history of liver disease associated with hepatitis C virus (HCV), with 3- to 5-fold higher odds of coinfected individuals developing cirrhosis. However, estimates of the seroprevalence of hepatitis C among people living with HIV/acquired immune deficiency syndrome (AIDS) (PLHA) in Latin America and the Caribbean (LAC) are widely variable.

**Methods:**

We performed a systematic review to estimate the seroprevalence of HCV among PLHA. We searched studies on HIV and HCV infections in LAC included in the PubMed, LILACS and Embase databases in December of 2014 with no time or language restrictions. The following combinations of search terms were used in the PubMed and Embase databases: (HIV OR Acquired Immunodeficiency Syndrome Virus OR AIDS OR HTLV OR Human Immunodeficiency Virus OR Human T Cell) AND (HCV OR HEPATITIS C OR HEPATITIS C VIRUS OR HEPACIVIRUS) AND (name of an individual country or territory in LAC). The following search terms were used in the LILACS database: (HIV OR AIDS OR Virus da Imunodeficiencia Humana) AND (HCV OR Hepatite C OR Hepacivirus). An additional 11 studies were identified through manual searches. A total of 2,380 publications were located, including 617 duplicates; the remaining articles were reviewed to select studies for inclusion in this study.

**Results:**

A total of 37 studies were selected for systematic review, including 23 from Brazil, 5 from Argentina, 3 from Cuba, 1 from Puerto Rico, 1 from Chile, 1 from Colombia, 1 from Mexico, 1 from Peru and 1 from Venezuela. The estimated seroprevalence of HCV infection varied from 0.8 to 58.5 % (mean 17.37; median 10.91), with the highest in Argentina and Brazil and the lowest in Venezuela and Colombia.

**Conclusions:**

Investigation of HCV infection among PLHA and of HIV infection among people living with HCV is highly recommended because it allows for better follow up, counseling and treatment of HIV/HCV-coinfected patients. Future studies with larger sample sizes are needed in both South and Central America to understand and address the risk factors associated with the acquisition of infection.

**Electronic supplementary material:**

The online version of this article (doi:10.1186/s12879-016-1988-y) contains supplementary material, which is available to authorized users.

## Background

Approximately 2.2 to 3.0 % of the world’s population (130–170 million people) is infected with the hepatitis C virus C (HCV), and approximately 36.7 million people live with human immunodeficiency virus infection/acquired immune deficiency syndrome (HIV/AIDS) [1, 2]. The occurrence of coinfection has been reported because HCV and HIV share the same transmission mechanisms.

Although HIV/HCV-coinfected individuals do not seem to have an increased risk of AIDS, kidney disease or heart disease, their odds of developing cirrhosis are higher [3]. HCV infection increases the number of deaths due to liver disease among coinfected individuals but does not influence the virological or immunological responses to highly active antiretroviral therapy (HAART) [4].

There is evidence that HIV may negatively influence the progression of HCV-related liver disease. According to one meta-analysis [5], the prevalences of cirrhosis in populations of HIV-infected individuals 20 and 30 years after HCV infection were 21 % (95 % CI: 16–28 %) and 49 % (95 % CI: 40–59 %), respectively. Other studies [6, 7] found that HIV-induced immunosuppression accelerated the natural history of HCV-related liver disease and that the odds of coinfected patients developing cirrhosis were 3- to 5-fold higher.

Additionally, the odds of hepatotoxicity due to HAART are higher among HIV/HCV coinfected patients than in individuals with HIV monoinfection [8].

In contrast to the situation in Europe and the United States, few data are available concerning HCV/HIV coinfection in Latin America and the Caribbean (LAC) despite their relevance for the formulation of public health policies. The aim of the present study was to investigate the seroprevalence of HCV infection among people living with HIV/AIDS (PLHA) in LAC.

## Methods

We performed a systematic review of published studies on the seroprevalence of HCV infection among PLHA in countries and/or territories in LAC. The review was performed and described following the “PRISMA” (Preferred Reporting Items for Systematic Reviews and Meta-Analysis) Statement published in 2009 [9].

### Search strategies

We searched all studies on HIV and HCV infection in LAC included in the PubMed, LILACS (Literatura Latino-Americana e do Caribe em Ciências da Saúde/Latin American and Caribbean Health Sciences Literature) and Embase databases in December of 2014 with no time or language restrictions. The following combinations of search terms were used in the PubMed and Embase databases: (HIV OR Acquired Immunodeficiency Syndrome Virus OR AIDS OR HTLV OR Human Immunodeficiency Virus OR Human T Cell) AND (HCV OR HEPATITIS C OR HEPATITIS C VIRUS OR HEPACIVIRUS) AND (name of an individual country or territory in LAC). The following search terms were used in the LILACS database: (HIV OR AIDS OR Virus da Imunodeficiencia Humana) AND (HCV OR Hepatite C OR Hepacivirus). The keywords were used as “text” (all fields) in the databases. We performed a manual search of the references cited in the selected studies and review articles to detect additional relevant publications. All instances of disagreement in the identification of relevant publications were discussed until a consensus was reached. To achieve this consensus, the researchers responsible for each phase review spoke either in person or by phone to present their arguments. If the disagreement persisted, the references in question were selected for the next phase of the study.

The titles and abstracts of the located publications were independently analyzed by 2 examiners (KYI and BPD), resulting in a list of potentially relevant studies. The full texts of these articles were analyzed for inclusion in the systematic review.

Articles describing data on HIV/HCV coinfection with a serologic diagnosis of HIV and HCV infection that reported estimates of the prevalence of anti-HIV/anti-HCV antibodies among HIV-infected individuals were included in the review.

### Study selection

We included original articles reporting the seroprevalence of antibodies against HCV (anti-HCV) among PLHA in LAC in the review provided that the number of participants was 50 or larger. We did not include case reports, case series, review articles, comments, or studies whose participants did not reside in LAC or had been described in previous publications. In the case of multiple studies performed on the same population, only the most complete data were included in the study. We also excluded self-reported HIV and/or HCV infection, data resulting from mandatory reporting of HIV and/or HCV infection (e.g., databases of national health ministries), specific groups of PLHA (e.g., drug users and homeless) and data from clinical trial or therapeutic studies.

The following definitions were used in the present review: (1) HIV infection: presence of anti-HIV antibodies based on immunoenzymatic methods; (2) HCV infection: presence of anti-HCV antibodies based on the immunoenzymatic method or immunoblotting; and (3) LAC: the following countries and territories - Argentina, Bolivia, Brazil, Chile, Colombia, Ecuador, French Guiana, Guyana, Paraguay, Peru, Suriname, Uruguay, Venezuela, Belize, Costa Rica, El Salvador, Guatemala, Honduras, Mexico, Nicaragua, Panama, Aruba, Antigua and Barbuda, Aruba, Bahamas, Barbados, Bonaire, British Virgin Islands, Cayman Islands, Cuba, Curaçao, Dominica, Dominican Republic, Grenada, Guadalupe, Haiti, Jamaica, Martinique, Montserrat, Puerto Rico, Saba, Saint Barthélemy, Saint Kitts and Nevis, Saint Lucia, Saint Martin, Saint Vincent and the Grenadines, Sint Eustatius, Sint Maarten, Trinidad and Tobago, Turks and Caicos Islands and the United States Virgin Islands.

### Data extraction

The data were independently collected by 2 examiners (MCM and CM); instances of disagreement were solved by discussion and consensus. The following data were extracted from the selected articles: author, year of publication, country, period of data collection, type of study, investigated population, sample size, average age, participants’ genders, seroprevalence of HCV, and method used to establish the hepatitis C diagnosis.

Some articles did not report all of the seroprevalence-related variables; in these cases, the missing data were calculated based on the reported values (e.g., the numerator was calculated from the reported denominator and seroprevalence).

### Assessment of the quality of the studies

Based on the criteria formulated by Boyle [10], Fowkes & Fulton [11], Loney [12] and Prins [13], we elaborated a list of criteria to assess the adequacy of the following aspects: sampling (11 items: study design, prospective data collection, definition of the target population, probabilistic sampling, sample size calculation, inclusion and exclusion criteria, specified data collection period, specified age variation, participant selection, acceptable losses, and representative sample), data collection (4 items: standardized data collection, clear defined outcomes, clear description of the outcome detection method, and valid method for outcome diagnosis), and data analysis and description (6 items: description of statistical analysis, total number of participants, number of events (outcome), prevalence by age and gender, prevalence including confidence intervals, and satisfactory confidence intervals). The total number of items was 21. The items were scored as positive or negative without relevance weighting. Larger scores (positive responses) corresponded to studies with better quality relative to the aims of the present review. The quality of the studies was independently assessed by 2 examiners (KYI and BPD).

## Results

**Fig. 1:**
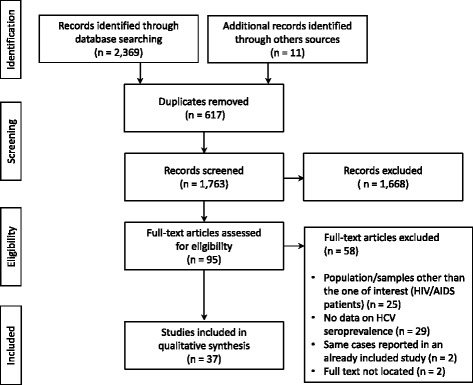
Flowchart of the identification, inclusion, and exclusion of the studies

A total of 2,369 articles was located in the investigated databases (PubMed, LILACS and Embase), and an additional 11 articles were identified through a manual search of the references cited in the selected studies and review articles (Fig. 1). Following the exclusion of duplicates (617), 1,753 articles remained for the abstract analysis. Review of the abstracts led to the exclusion of 1,668 articles. Thus, 95 articles were selected for the full-text analysis, of which 37 (*n* = 21,383 individuals) published from 1992 to 2014 were included in the systematic review.

Among these 37 studies on the seroprevalence of HCV infection among PLHA [14–50], 23 studies were conducted in Brazil, 5 in Argentina, 3 in Cuba, 1 in Puerto Rico, 1 in Chile, 1 in Colombia, 1 in Mexico, 1 in Peru and 1 in Venezuela (Table 1). The sample size varied from 60 to 2,994. Prospective data collection was performed in 16 studies (43.24 %). Diagnosis of HCV infection was established based on anti-HCV antibody detection using enzyme-linked immunosorbent assay (ELISA) 3 in 15 studies (40.54 %), ELISA2 in 3 studies (8.11 %), ELISA1 in 1 study (2.70 %) and (unspecified) ELISA in 18 studies (48.69 %). The average quality score was 10.76 (variation: 4–18), with 7 studies scoring 7 points or less, 26 scoring 8 to 14 points and 4 scoring 15 to 21 points. The most common cause of impaired quality scores was the methods used for sampling (more detailed data on the assessment of the quality of studies are available in the Additional files 1 and 2).

**Table 1:** Seroprevalence studies of hepatitis C virus (HCV) in people living with HIV/AIDS in Latin America and the Caribbean

Author	Year	Location	Gender	Mean age (years)	Data collection	Sample size	Anti-HCV detection *N* (%)	ELISA kit generation
Freitas [14]	2014	BRAZIL	M/F	41.6	P	848	59 (7.0 %)	NA
Librelotto [15]	2014	BRAZIL	M/F	41.9	R	148	16 (10.8 %)	NA
Jaspe [16]	2014	VENEZUELA	M/F	NA	R	418	3 (0.7 %)	3th generation
Simon [17]	2014	BRAZIL	M/F	40.6	P	580	138 (23.8 %)	3th generation
Távora [18]	2013	BRAZIL	M/F	NA	R	329	23 (7.0 %)	NA
Brunetta [19]	2013	BRAZIL	M/F	NA	R	701	146 (20.8 %)	NA
Farias [20]	2013	ARGENTINA	M/F	39.2	R	238	62 (26.0 %)	NA
Oliveira-Filho [21]	2012	BRAZIL	M/F	38.1	R	768	52 (6.8 %)	3th generation
Victoria [22]	2010	BRAZIL	M/F	38.5	R	1,582	70 (4.4 %)	NA
Wolff [23]	2010	BRAZIL	M/F	40.3	R	1,143	357 (31.2 %)	3th generation
Guimarães [24]	2010	BRAZIL	M/F	41.6	R	110	10 (9.1 %)	3th generation
Santos [25]	2010	BRAZIL	M/F	33.8	R	250	78 (31.2 %)	NA
Perez [26]	2010	PUERTO RICO	M/F	NA	P	1,650	86 (5.2 %)	NA
Sampaio [27]	2009	BRAZIL	M/F	39.3	P	429	46 (10.7 %)	3th generation
Pérez [28]	2009	CHILE	M/F	40.9	R	273	7 (2.6 %)	NA
Carvalho [29]	2009	BRAZIL	M/F	NA	P	343	14 (4.1 %)	3th generation
Ré [30]	2008	ARGENTINA	M/F	NA	P	310	39 (12.6 %)	NA
Carmo [31]	2008	BRAZIL	M/F	NA	R	824	76 (9.2 %)	3th generation
Reiche [32]	2008	BRAZIL	M/F	NA	P	757	159 (21.0 %)	NA
dos Santos [33]	2008	BRAZIL	M/F	NA	P	299	10 (3.3 %)	3th generation
Alfonso [34]	2008	CUBA	M/F	NA	R	90	18 (20.0 %)	NA
Mussi [35]	2007	BRAZIL	M/F	37.2	P	1,008	110 (10.9 %)	NA
Quarleri [36]	2007	ARGENTINA	M/F	39	P	593	129 (21.8 %)	3th generation
Rivas-Estilla [37]	2007	MEXICO	M/F	34	P	140	17 (12.1 %)	3th generation
Hoyos-Orrego [38]	2006	COLOMBIA	M/F	37.9	P	251	2 (0.8 %)	2th generation
Tovo [39]	2006	BRAZIL	M/F	34.4	R	330	126 (38.2 %)	3th generation
de Carvalho [40]	2006	BRAZIL	M/F	NA	P	343	14 (4.1 %)	3th generation
Bello Corredor [41]	2005	CUBA	NA	NA	R	2,994	314 (10.4 %)	NA
Rodriguez [42]	2005	CUBA	F	29	R	60	5 (8.4 %)	NA
de los Angeles Pando [43]	2004	ARGENTINA	M/F	36.7	R	165	53 (30.5 %)	3th generation
Segurado [44]	2004	BRAZIL	M/F	NA	P	495	179 (36.2 %)	3th generation
Pavan [45]	2003	BRAZIL	M/F	30.8	P	232	119 (53.8 %)	2th generation
Mendes-Corrêa [46]	2001	BRAZIL	M/F	34.08	R	1,457	258 (17.7 %)	2th generation
Treitinger [47]	1999	BRAZIL	NA	NA	R	93	50 (53.8 %)	NA
Fainboim [48]	1999	ARGENTINA	M/F	29	P	484	283 (58.5 %)	NA
Edelenyi-Pinto [49]	1993	BRAZIL	M/F	NA	R	187	28 (15.0 %)	1th generation
Hyams [50]	1992	PERU	M/F	NA	R	305	13 (4.3 %)	NA

*M* male, *F* female, *P* prospective, *R* retrospective, *NA* not available

The estimated seroprevalence of HCV infection in the 37 selected studies from the LAC region varied from 0.7 to 58.5 % (mean 17.4; median 10.9) (Table 1). The highest prevalence was found in studies from Argentina and Brazil and the lowest from Venezuela and Colombia (Table 2).

**Table 2:** Seroprevalence of HCV infection in the general population and PLHA by country

Country	Seroprevalence of HCV in general population [1, 63] (%)	Seroprevalence^a^ of HCV in HIV patients (%)
Argentina	1.9	29.9
Brazil	1.4	18.9
Chile	0.8	2.6
Colombia	1.0	0.8
Cuba	1.8	12.9
Mexico	1.0	12.1
Peru	1.0	4.3
Puerto Rico	6.3	5.2
Venezuela	0.9	0.7

^a^Mean seroprevalence found in the selected studies

**Fig. 2:**
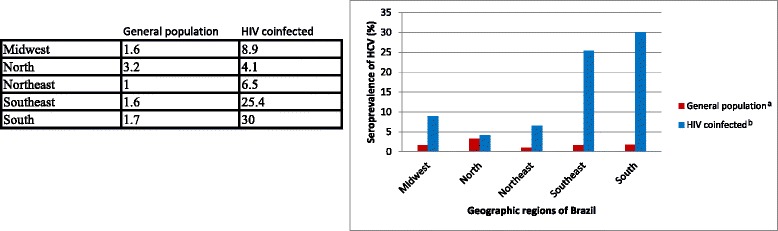
Seroprevalence of HCV in the general population and in PLHA by geographic region in Brazil. Footnote of Fig. 2: ^a^Based in Pereira et al. [63]; ^b^Median of the HCV seroprevalence found in the selected studies

Most of the selected studies were performed in Brazil. Figure 2 shows the geographic distribution of PLHA coinfected with HCV in Brazil described in these studies. Notably, the southern region had 30 % of all cases [15, 17, 23, 25, 32, 39, 47], followed by the southeast (25.4 %) [19, 24, 44–46, 49] and midwest (8.9 %) [14, 35] regions (Fig. 2).

## Discussion

HCV infection is a significant cause of increased morbidity and mortality among individuals living with HIV/AIDS and other populations of immunodeficient patients as a whole. If we consider that 1 out of 10 individuals living with HIV/AIDS also has hepatitis C, there are 175,000 HIV/HCV coinfected individuals in LAC (based on 1,750,000 individuals living with HIV/AIDS). By summarizing the results of several studies, we attempted to present a comprehensive summary of the literature on the subject and to gain a new understanding of the distribution of anti-HCV among PLHA.

Our systematic review of the prevalence of hepatitis C among individuals living with HIV/AIDS included reports from 37 studies corresponding to 21,383 participants residing in the LAC region. Our main findings included the wide heterogeneity in the information concerning the subject of interest, with the relevant studies conducted in only 9 countries, most of which were in South America. Our review showed that the mean LAC regional seroprevalence of hepatitis C among PLHA was approximately 17.4 %. The substantial heterogeneity observed in our study suggests that caution is required when pooled estimates are used. Because an error can occur in the estimated prevalence when attempting to perform a meta-analysis of very heterogeneous data, a meta-analysis was not performed. Additionally, our findings emphasize the need for surveys to include careful descriptions of the sampling procedures and diagnostic methods.

We were not able to locate any review studies on the seroprevalence of hepatitis C among PLHA specifically in the LAC region. In a large cohort of primarily European HIV-infected individuals, Rockstroh et al. found that 33 % of the patients also exhibited HCV infection. Approximately 25 % of the participants were injection drug users (IDUs). The proportion of IDUs among individuals with hepatitis C was 77.5 %. In one study conducted in HIV-infected patients in the United States [51], 16 % of the sample also exhibited HCV infection. Approximately 20 % of the participants were IDUs, of which 72.7 % were HCV-positive. In one study performed in Russia, 91 % of HIV-infected IDUs exhibited anti-HCV antibodies [52]. Similarly, high rates of HIV/HCV coinfection were found in the United States [53], Australia [54], India [55], northern Vietnam [56] and some regions in China [57, 58]. In the Swiss HIV Cohort Study, the prevalences of HIV/HCV coinfection among IDUs and homosexual and heterosexual men were 87.7, 3.7 and 6 %, respectively [59]. An association between drug use and HIV/HCV coinfection was reported in several studies [51, 59, 60].

One possible cause for the heterogeneity in the seroprevalence results extracted from the selected studies may be differences in the proportion of IDUs included in the studies; we could not analyze this factor with the available data. According to Nelson et al. [61], the prevalence of HCV infection among IDUs in Latin America is approximately 67 %, with a range from 10 to 97 %. These authors collected data corresponding to 5 countries [Argentina (54.6 %), Brazil (63.9 %), Mexico (97.4 %), Paraguay (9.8 %) and Uruguay (21.9 %)] but were unable to locate sufficient information to estimate the HCV prevalence in the Caribbean.

The seroprevalence results reported in the studies selected in this systematic review may also be attributed to the different prevalence rates of HCV among the overall population in the locations where the studies were conducted. It is possible to speculate that the HCV prevalence in target populations (here, PLHA) is higher in locations where the prevalence of HCV among the overall population is also higher, although not all studies selected for this review support this hypothesis. This possibility is a cause for concern because the seroprevalence data included in our review do not include the locations with the highest hepatitis C prevalence in the LAC region, such as Bolivia (4.7 %) and Haiti (4.4 %) [1]. The use of an ELISA method for anti-HCV detection was considered appropriate, but differences in the seroprevalence might be due to the kit used to establish the hepatitis C diagnosis because the tests might present certain differences in sensitivity and specificity. The third- or fourth-generation immunoenzymatic assays are considered better because they contain HCV core antigens and HCV nonstructural genes [62]. Therefore, third- and fourth-generation ELISAs are more specific diagnostic techniques. When these methods are used, the rate of positive results is lower than that obtained with older methods (first- and second-generation ELISAs).

The seroprevalence of HCV among PLHA seems to be higher than that in the overall population. This finding suggests that preventive measures targeting the overall population do not reduce the prevalence of HCV or HIV in the previously infected population (HIV or HCV). More direct comparisons should be performed with studies using the same sampling methods and techniques to establish the diagnosis of hepatitis C in both the overall population and the population of HIV-seropositive individuals.

A limitation of the present study is that the data collection was quite heterogeneous. Most studies used convenience samples, which may not be representative of the population of individuals living with HIV/AIDS. Additionally, we did not analyze the studies per country or territory because they were only conducted in 9 countries (6 in South America, 2 in Central America and 1 in North America).

## Conclusions

The high mean seroprevalence found in the LAC region (17.4 %) reinforced the recommendation that investigations of HCV infection among PLHA and of HIV infection among individuals with hepatitis C are highly recommended to allow for better follow up, counseling and treatment of HIV/HCV-coinfected patients. Future studies with larger sample sizes are needed in both South and Central America to understand and address the risk factors associated with the acquisition of infection.

## Additional files

Additional file 1: Instrument for assessment of the quality of the studies. Description: describes the items considered in the assessment of the quality of the studies. (DOCX 12 kb)
Additional file 2: Assessment of the quality of the studies Description: contains data corresponding to the quality of the studies that describe their (quality) total score relative to the following items: sampling process, procedures used for data collection, and data analysis and description. (DOC 82 kb)

## Abbreviations

AIDS: Acquired immune deficiency syndrome
Anti-HCV: Antibodies against HCV
ELISA: Enzyme-linked immunosorbent assay
HAART: Highly active antiretroviral therapy
HCV: Hepatitis C virus
HIV: Human immunodeficiency virus
IDU: Injection drug user
LAC: Latin America and the Caribbean
PLHA: People living with HIV/acquired immune deficiency syndrome
