# Supplementary material for: Seroprevalence of hepatitis C virus among people living with HIV/AIDS in Latin America and the Caribbean: a systematic review
Source: BMC Infect Dis. 2016 Nov 9;16:663. doi: 10.1186/s12879-016-1988-y (PMC5103446; doi:10.1186/s12879-016-1988-y)
Supplement: Additional file 1: — Instrument for assessment of the quality of the studies. Description: describes the items considered in the assessment of the quality of the studies. (DOCX 12 kb) [file 12879_2016_1988_MOESM1_ESM.docx]

**Additional file 1. Instrument for assessment of the quality of the studies**

**Title: .................................................................................................................................................**

**Authors: ............................................................................................................................................**

**Year: .................................................................................................................................................**

**Answer yes (Y) or no (N):**

1. Is the study design adequate for its aims ......................................................................................|__|

2. Were the data prospectively collected? ........................................................................................|__|

3. Was the target population clearly defined .....................................................................................|__|

4. Was probabilistic sampling used to identify potential participants? ..............................................|__|

5. Was the sample size calculated? ....................................................................................................|__|

6. Were the inclusion and exclusion criteria well defined? ................................................................|__|

7. Was the study period specified? ....................................................................................................|__|

8. Was the age variation specified? ....................................................................................................|__|

9. Was the selection of participants adequate? (Did the authors comply with the preset criteria?) . |__|

10. Is the proportion of non-participants adequate? Less than 30%? ................................................|__|

11. Is the sample of participants representative of the target population? .......................................|__|

12. Was the data collection standardized? ........................................................................................|__|

13. Was the outcome clearly defined .................................................................................................|__|

14. Is the outcome measure adequately described? (detection method?) ........................................|__|

15. Is the method used for outcome diagnosis valid ..........................................................................|__|

16. Was the data analysis described in a complete manner? .............................................................|__|

17. Was the total number of participants clearly reported? ..............................................................|__|

18. Was the number of individuals who experienced the outcome clearly reported? .......................|__|

19. Were the prevalence rates also described by age and sex? ..........................................................|__|

20. Did the authors report the 95% CI of the prevalence rates? .........................................................|__|

21. Are the reported CI satisfactory? .................................................................................................|__|

**Total score |____|**
