# Supplementary material for: Seroprevalence of hepatitis C virus among people living with HIV/AIDS in Latin America and the Caribbean: a systematic review
Source: BMC Infect Dis. 2016 Nov 9;16:663. doi: 10.1186/s12879-016-1988-y (PMC5103446; doi:10.1186/s12879-016-1988-y)
Supplement: Additional file 2: — Assessment of the quality of the studies Description: contains data corresponding to the quality of the studies that describe their (quality) total score relative to the following items: sampling process, procedures used for data collection, and data analysis and description. (DOC 82 kb) [file 12879_2016_1988_MOESM2_ESM.doc]

**Additional file 2. Assessment of the quality of the studies**

| **AUTHOR** | **SAMPLE** | **COLLECTION** | **PRESENTATION** | **TOTAL SCORE** |
| --- | --- | --- | --- | --- |
| Freitas SZ 2014 | 8 | 4 | 6 | 18 |
| Librelotto CS 2014 | 6 | 2 | 2 | 10 |
| Jaspe RC 2014 | 1 | 4 | 2 | 7 |
| Simon D 2014 | 9 | 4 | 3 | 16 |
| Távora LG 2013 | 5 | 2 | 2 | 9 |
| Brunetta DM 2013 | 3 | 2 | 2 | 7 |
| Farías AA 2013 | 8 | 4 | 3 | 15 |
| Oliveira-Filho AB 2012 | 6 | 4 | 3 | 13 |
| Victoria MB 2010 | 7 | 1 | 3 | 11 |
| Wolff FH 2010 | 5 | 4 | 3 | 12 |
| Laufer N 2010 | 8 | 1 | 2 | 11 |
| Pérez CC 2009 | 4 | 2 | 2 | 8 |
| Carvalho FH 2009 | 7 | 4 | 3 | 14 |
| Ré V 2008 | 5 | 3 | 4 | 12 |
| Carmo RA 2008 | 6 | 4 | 4 | 14 |
| Reiche EM 2008 | 4 | 4 | 3 | 11 |
| Bello Corredor M 2005 | 2 | 3 | 2 | 7 |
| Mussi AD 2007 | 5 | 3 | 3 | 11 |
| Quarleri JF 2007 | 6 | 3 | 3 | 12 |
| Rivas-Estilla AM 2007 | 7 | 4 | 4 | 15 |
| Hoyos-Orrego A 2006 | 6 | 4 | 3 | 13 |
| Tovo CV 2006 | 1 | 4 | 3 | 8 |
| Mayor AM 2006 | 4 | 4 | 4 | 12 |
| Rodriguez ME 2005 | 2 | 2 | 3 | 7 |
| de los Angeles Pando M | 5 | 4 | 5 | 14 |
| Segurado AC 2004 | 3 | 4 | 5 | 12 |
| Pavan MH 2003 | 4 | 4 | 3 | 11 |
| Mendes-Corrêa MC 2001 | 4 | 3 | 2 | 9 |
| Treitinger A 1999 | 2 | 2 | 2 | 6 |
| Fainboim H 1999 | 3 | 3 | 2 | 8 |
| Edeleny-Pinto M 1993 | 2 | 4 | 2 | 8 |
| Hyams KC 1992 | 5 | 4 | 3 | 12 |
| Santos EO 2008 | 8 | 4 | 2 | 14 |
| Guimarães RO 2010 | 4 | 1 | 1 | 6 |
| Santos KF 2010 | 0 | 2 | 2 | 4 |
| Soares Sampaio A 2009 | 5 | 4 | 2 | 11 |
| Padron Alfonso A 2008 | 3 | 4 | 3 | 10 |
| Carvalho FHP 2006 | 3 | 3 | 3 | 9 |
| Perez CM 2010 | 7 | 4 | 3 | 14 |
